# The ColM Family, Polymorphic Toxins Breaching the Bacterial Cell Wall

**DOI:** 10.1128/mBio.02267-17

**Published:** 2018-02-13

**Authors:** Maarten G. K. Ghequire, Susan K. Buchanan, René De Mot

**Affiliations:** a Centre of Microbial and Plant Genetics, KU Leuven, Heverlee, Belgium; b Laboratory of Molecular Biology, National Institute of Diabetes and Kidney Diseases, National Institutes of Health, Bethesda, Maryland, USA; University of Nebraska—Lincoln

**Keywords:** bacteriocin, colicin M, diversifying recombination/selection, lipid II, periplasm, toxin-immunity module

## Abstract

Bacteria host an arsenal of antagonism-mediating molecules to combat for ecologic space. Bacteriocins represent a pivotal group of secreted antibacterial peptides and proteins assisting in this fight, mainly eliminating relatives. Colicin M, a model for peptidoglycan-interfering bacteriocins in Gram-negative bacteria, appears to be part of a set of polymorphic toxins equipped with such a catalytic domain (ColM) targeting lipid II. Diversifying recombination has enabled parasitism of different receptors and has also given rise to hybrid bacteriocins in which ColM is associated with another toxin module. Remarkably, ColM toxins have recruited a diverse array of immunity partners, comprising cytoplasmic membrane-associated proteins with different topologies. Together, these findings suggest that different immunity mechanisms have evolved for ColM, in contrast to bacteriocins with nuclease activities.

## PERSPECTIVE

Bacteriocin production represents a proficient bacterial strategy to eliminate phylogenetically related rivals, competing for nutrients and space. These ribosomally encoded antibacterial peptides and proteins, diverse in size—ranging from peptides to large multiprotein complexes—and mode of action, are ubiquitous and fulfill a pivotal role in colonizing different niches such as rhizosphere and gut (1–4). Bacteriocins identified in several proteobacterial genera, including *Escherichia*, *Pseudomonas*, and *Yersinia*, are also structurally very diverse, and a major subset of these molecules adopt a modular organization characteristic of polymorphic toxins. They consist of a receptor-binding domain (RBD) allowing docking onto the surface of a target cell, a moiety enabling transfer across the membrane, and a variable carboxy-terminal toxin domain mediating killing by different mechanisms (5–9).

Bacteriocinogenic strains need to impede the lethal intracellular activity of the toxins that they produce. For nuclease bacteriocins acting in the cytoplasm, this is achieved via coexpression of dedicated immunity genes encoding small proteins (80 to 160 amino acids [aa]) that attach to cognate toxin modules with strict specificity and high affinity (7, 10). Upon contact with target cells, this one-on-one protein association is lost (11, 12). For bacteriocins acting at the periplasmic level, such as pore formers and certain enzymes interfering with peptidoglycan metabolism, an integral membrane or membrane-anchored protein transiently impedes the bacteriocin’s action during secretion by (an) unknown mechanism(s). Colicins from *Escherichia coli* (8) and S-type pyocins from *Pseudomonas aeruginosa* (6) currently serve as model systems in Gram-negative bacteria for studying immunity functions, toxin activities, receptor interactions, and bacteriocin-import processes.

### Enzymatic attack on the bacterial inner fortress wall.

Colicin M from *E. coli* is one of the smallest modular bacteriocins studied to date. Its carboxy-terminal catalytic domain (ColM) displays phosphatase activity and cleaves the peptidoglycan building block lipid II, accessible from the periplasm. This leads to accumulation of undecaprenol (C_55_-OH) and 1-pyrophospho-MurNAc (*N*-acetylmuramic acid)-GlcNAc (*N*-acetylglucosamine), with MurNAc carrying the stem pentapeptide, which cannot be reused for murein biosynthesis (13–15). This enzymatic activity distinguishes the “protein antibiotic” ColM from antibiotics of different chemical classes acting on lipid II (16), and pesticin, which cleaves the glycan chain of murein (17). The recently described streptococcal LXG toxin TelC displays similar lipid II-degrading activity as ColM but adopts a different fold (18, 19).

ColM domain-encoding bacteriocin genes have also been characterized in *Burkholderia* (20), *Pectobacterium* (21), and *Pseudomonas* (22–25), here termed ColM-type burkhocins, pectocins, and pseudocins, respectively (Table 1). Such putative bacteriocin genes are also found in other genera, mainly in the *Enterobacteriaceae* (e.g., *Enterobacter*, *Klebsiella*, *Salmonella*, and *Serratia*). Notably, whereas colicin M genes are plasmid encoded in *E. coli* and some other *Enterobacteriaceae* (such as *Citrobacter* and *Shigella*), usually joined upstream by a colicin B pore-former–immunity gene tandem (26), ColM-type bacteriocin genes in other genera reside on chromosomes, often within or in close proximity to a mobile context or regions prone to recombination events, such as tailocin units, genomic islands, and transposases (21, 22, 24, 27).

**TABLE 1:** Summary of functionally characterized bacteriocins equipped with a ColM domain^f^

ColM type	Species	Strain	Bacteriocin			ColM immunity protein				Replicon	Receptor
			Name	Size (aa)	Domain(s)	Type	Size (aa)	Domain	*imm* position^e^		
ColMα	*Escherichia* *coli*	SMS-3-5 (pSMS35_130)	Colicin M	271	PF14859^a^	Cmi	117	PF13995^d^	D (−)	Plasmid	FhuA
	*Pectobacterium* *carotovorum*	PC1	Pectocin M1	268	PF00111,^b^ PF14859	Cmi*	117	PF13995	D (−)	Chromosome	FusA
		PBR1692	Pectocin M2	271	PF00111, PF14859	Cmi*	123	PF13995	?	Chromosome	FusA*
		JJ692	Pyocin M1 (PaeM)	289	PF14859	PmiA*	142	ND	D (+)	Chromosome	FiuA*
	*Pseudomonas* *aeruginosa*	NCTC10332	Pyocin M1 (PaeM)	289	PF14859	PmiA	142	ND	D (+)	Chromosome	FiuA
		BL03	Pyocin M4 (PaeM4)	342	PF14859	PmiC*	232	ND	D (−)	Chromosome	?
	*Pseudomonas* *fluorescens*	Q8r1-96	PflM	271	PF14859	PmiA	142	ND	D (+)	Chromosome	FiuA
	*Pseudomonas* *synxantha*	BG33R	PmnH	462	PF14859, PF01024^c^	?	?	?	?	Chromosome	FiuA
	*Pseudomonas* *syringae*	DC3000	Syringacin M (PsyM/SyrM)	276	PF14859	PmiA	135	ND	D (+)	Chromosome	FiuA*
											
ColMβ	*Burkholderia* *ambifaria*	MEX-5	Burkhocin M1 (BurM1)	379	PF14859	BmiA	110	ND	U (−)	Chromosome	?
		AMMD	Burkhocin M2 (BurM2)	372	PF14859	BmiB	124	ND	D (+)	Chromosome	?

a PF14859, colicin M (ColM).

b PF00111, Fer2 (2Fe-2S iron-sulfur cluster binding domain).

c PF01024, colicin (colicin pore-forming domain).

d PF13995, YebF (YebF-like protein).

e Immunity gene (*imm*) positions: D, downstream; U, upstream; +, same strand; −, opposite strand.

f Putative immunity partners and/or receptors are marked with an asterisk. ND, not detected; ?, unknown. Pyocin M1 proteins from JJ692 and NCTC10332 share 90% pairwise amino acid identity. PFAM families are indexed a-b-c-d.

The catalytic mechanism of ColM-mediated lipid II degradation remains elusive, although mutagenesis studies with *E. coli* and *P. aeruginosa* ColM bacteriocins allowed the identification of a DXYDX_5_HR motif required for the hydrolase function (14, 28). Some degeneracy in this signature sequence appears to exist, e.g., in the ColM-type burkhocin from *Burkholderia ambifaria* MEX-5 as DXFKX_5_R (see Fig. S1 in the supplemental material). For this protein, phosphatase activity via a lipid II hydrolase assay remains to be assessed (20). Interestingly, even in a mutated pectocin M1 protein where lipid II degradation is abolished, the ColM module may still provoke cellular lysis when secreted to the periplasm, indicating that inhibition by ColM domains may not be based solely on the known catalytic activity (29). This is reminiscent of the collateral damage to membranes and membrane-bound complexes that can be caused by lipid II-binding antibiotics (16). Significant variation in the relative positioning of the side chains of the ColM signature residues has been observed as well (Fig. S2) (23, 30–32). Such structural flexibility suggests reorientation of these key residues to accommodate binding of lipid II and to act in a concerted way (23, 31, 32). ColM hydrolase activity does not seem to be highly specific, since cleavage of peptidoglycan precursors from Gram-positive bacteria was noted too (31, 33).

10.1128/mBio.02267-17.1 FIG S1  Multiple sequence alignment of ColM domains. One representative bacteriocin per genus was selected in the case of highly homologous sequences (>80% pairwise amino acid identity for full-length proteins). Gray shading reflects the degree of sequence conservation. The ColM signature motif (consensus DXYDX_5_HR) is highlighted in red. The partially conserved proline residue likely isomerized by FpkA is colored blue. Abbreviations: Acat, *Acidovorax cattleyae*; Bamb, *Burkholderia ambifaria*; Bcep, *Burkholderia cepacia*; Bglu, *Burkholderia glumae*; Bokl, *Burkholderia oklahomensis*; Brgoo, *Brenneria goodwinii*; Bsp, *Burkholderia* sp.; Bubo, *Burkholderia ubonensis*; Cfre, *Citrobacter freundii*; Dchr, *Dickeya chrysanthemi*; Ecol, *Escherichia coli*; Enaer, *Enterobacter aerogenes*; Enkob, *Enterobacter kobei*; Ermal, *Erwinia mallotivora*; Ersp, *Erwinia* sp.; Ilim, *Inquilinus limosus*; Kpne, *Klebsiella pneumoniae*; Kvar, *Klebsiella variicola*; Lyeo, *Luteibacter yeojuensis*; Paana, *Pantoea ananatis*; Pacon, *Pantoea conspicua*; Paer, *Pseudomonas aeruginosa*; Pbre, *Pseudomonas brenneri*; Pced, *Pseudomonas cedrina*; Pecar bra/car, *Pectobacterium carotovorum* subsp. *brasiliense*/*carotovorum*; Pflu, *Pseudomonas fluorescens*; Pput, *Pseudomonas putida*; Psp, *Pseudomonas* sp.; Psyr (ace/tom/mor), *Pseudomonas syringae* (pv. aceris/tomato/morsprunorum); Sent (ent), *Salmonella enterica* (subsp. *enterica*); Semar, *Serratia marcescens*; Shboy, *Shigella boydii*; Stmal, *Stenotrophomonas maltophilia*. Download FIG S1, TIF file, 7.1 MB. Copyright © 2018 Ghequire et al. 2018 Ghequire et al. This content is distributed under the terms of the Creative Commons Attribution 4.0 International license.

10.1128/mBio.02267-17.2 FIG S2  Cartoon representation of colicin M (PDB 2XMX) (A), PaeM (PDB 4G75) (B), and pectocin M2 (PDB 4N58) (C). ColM domains are shown in wheat color; the FhuA- and FiuA-targeting sequences from colicin M and PaeM, respectively, are colored in blue; amino-terminal parts comprising the TonB box are shown in lemon color; the FusA-targeting ferredoxin module in pectocin M2 is dark green; and the helix connecting the ferredoxin module and ColM domain in pectocin M2 is dark gray. ColM domains require the presence of a coordinating divalent cation for catalytic functionality (Mg^2+^ shown as olive sphere; detected only in the structure of PaeM). The ferredoxin module binds 2Fe-2S (yellow). Side chains of residues constituting the ColM motif are shown as black sticks. The proline residue (P176) likely targeted by FkpA in colicin M is indicated. Download FIG S2, TIF file, 0.8 MB. Copyright © 2018 Ghequire et al. 2018 Ghequire et al. This content is distributed under the terms of the Creative Commons Attribution 4.0 International license.

### Polyphyletic nature of the enzyme module.

**FIG 1:**
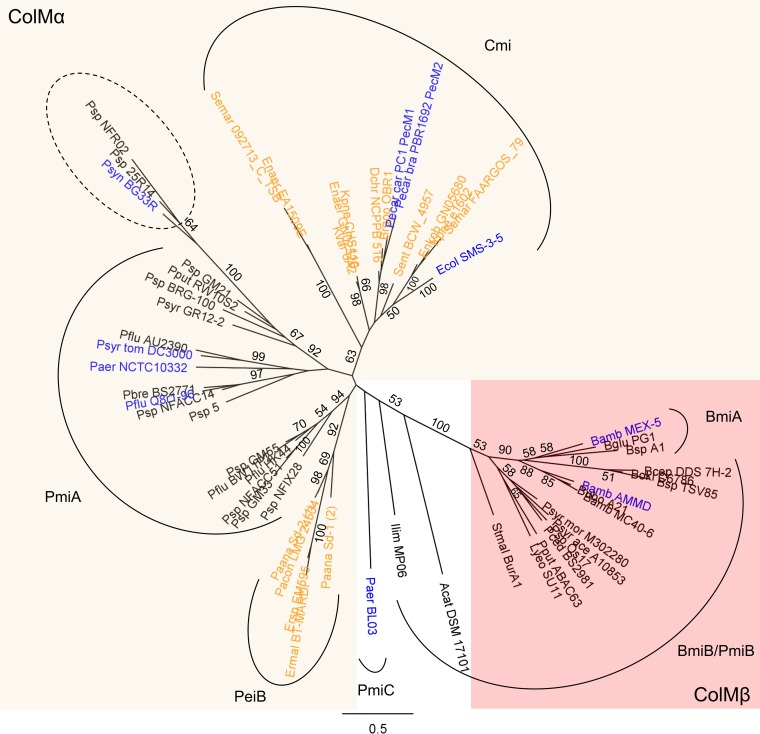
Phylogeny of ColM domains derived from characterized and putative bacteriocins. Maximum likelihood phylogenetic tree (PhyML, JTT substitution model) of ColM domains of selected bacteriocins: highly homologous sequences per genus (>80% pairwise amino acid identity for full-length proteins) are represented by one sequence only. Bootstrap values (percentages of 1,000 replicates) higher than 50 are shown at the branches; the scale bar represents 0.5 substitutions per site. ColMα-type, ColMβ-type, and ColMs with unassigned positioning are shown on a red, a wheat-color, and a white background, respectively. Functionally characterized ColM bacteriocins are marked in blue, and other enterobacterial ColMs are marked in orange. Species abbreviations are defined in the legend to Fig. S1. *Citrobacter freundii* AMA 948, *Shigella boydii* 1221, *Klebsiella pneumoniae* k1004, and *Salmonella enterica* subsp. *enterica* DT104 encode a ColM bacteriocin highly homologous to colicin M from *Escherichia coli* (~99% pairwise amino acid identity) and are not displayed at the node of “Ecol SMS-3-5” for legibility. ColM domains that are part of a hybrid bacteriocin with dual toxin architecture are grouped by a dashed ellipse. Enterobacterial ColM domains from the Cmi-joined clade share ~53% pairwise amino acid identity; more remote members in *Pantoea* and *Erwinia* share lower amino acid identities with their enterobacterial counterparts (23 to 42% pairwise amino acid identity). ColMs from pseudomonads in the ColMα clade share ~37% pairwise amino acid identity. The type of (putative) immunity partner for ColM—encoded downstream or upstream or separated from the bacteriocin—is displayed next to the clade (delineated by arcs).

Phylogenetic analysis of ColM domains (Pfam PF14859) extracted from characterized bacteriocins and homologues reveals that, with few exceptions, they are affiliated with one of two major clades, here termed ColMα and ColMβ (Table 1; Fig. 1). In addition to *E. coli* and a number of enterobacterial species, ColMα domain representatives occur in several other gammaproteobacteria, including pectobacteria producing the ColM-type pectocins (21) and pseudomonads. In the latter genus, a large number of diverged ColMα domains can be discerned, reflecting its enormous phylogenomic divergence (34), but the ColM signature motif can be recognized well in most catalytic domains (Fig. S1). Two notable exceptions are those in which the ColM domain is not present at the carboxy terminus. In *Pseudomonas synxantha* BG33R and about 10 other strains, the ColM domain (motif HXYDX_5_FK) is fused at the C terminus with a ColN-related pore-forming domain (6, 35, 36). Bacteriocin functionality of this hybrid toxin, termed PmnH, has been demonstrated, although the exact contribution of the ColM domain in the antibacterial function remains elusive (37). Conceivably, the recruitment of a second killing module would offer a competitive advantage for producer strains since cellular death may be circumvented only when targeted strains express two compatible immunity proteins. For another hybrid bacteriocin organization found in *Pseudomonas* sp. strain NFR02, the ColM domain is followed by a pyocin S domain (Pfam PF06958)—involved in membrane translocation of numerous nuclease bacteriocins (38)—and a toxin-immunity module previously identified in carocin D (no Pfam domain assigned) (39). The latter killer unit acts as a DNase but is distinct from HNH- and non-HNH-type DNases (7, 10, 40). Significant deviations from the ColM motif (IXYNX_5_LK) in this second hybrid (tentatively named PmdH) suggest that in this protein the phosphatase function is abrogated (Fig. S1).

The ColMβ clade is populated by fewer representatives, mainly originating from *Burkholderia* and *Pseudomonas*, with some *Pseudomonas* strains carrying bacteriocin genes of both types, i.e., a ColMα- and a ColMβ-type gene, at unlinked locations (for example, *Pseudomonas syringae* pv. morsprunorum M302280) (24). No crystal structure is available yet for a ColMβ representative. An interesting observation is that ColM-type burkhocins are consistently equipped with a short carboxy-terminal extension of ~30 aa (20), not part of the ColM module. The exact role of this extra segment remains elusive, though it likely does not contribute to the hydrolase or immunity function (see below), given its absence in ColMβ-type bacteriocins from *Pseudomonas*, *Stenotrophomonas*, and *Luteibacter*.

### Different routes for bacteriocin release.

Bacteriocin secretion is a costly trait for producer strains since this step requires cell lysis. Colicins are generally released to the medium following expression of a lysis gene in close proximity to the bacteriocin genes. However, such a gene is not present near *colM* in *Escherichia coli*, nor is this the case for chromosome-carried *colM* genes (8, 41). This raises the question of how these compounds can be secreted by the cell. Earlier, it was demonstrated that colicins lacking an adjacent lysis module may take advantage of a lytic cassette of a prophage encoded elsewhere in the genome (42), and similar secretion “piggybacking” via prophage or tailocin clusters has been suggested for other midsize bacteriocins as well (27). Given the high cost of lytic release, only a fraction of a total cell population is therefore sacrificed for compound release, as demonstrated for a colicin A-E2-E7 system (43). Among ColM-type bacteriocins, only the burkhocins are preceded by a cleavable secretion signal sequence (20), indicative of membrane passage via the Sec pathway. This was previously observed for other proteobacterial bacteriocin systems as well, such as certain lectin-like bacteriocins (44, 45). It remains unclear, however, what evolutionary drivers determined the incorporation of signal sequences and what environmental cues trigger ColM-type bacteriocin expression.

### A distinct polymorphic toxin family generated by diversifying recombination.

Initiation of import of modular bacteriocins, and hence specificity, depends on the presence of specific outer membrane proteins (OMPs) exposed at the cellular surface. Colicin M anchors to (certain loops of) the ferrichrome transporter FhuA and utilizes this OMP for translocation into the periplasmic space (Table 1) (17, 46). This transfer proceeds through the protein barrel, mediated by the TonB-ExbB-ExbD complex, is energized by the proton motive force and likely requires (partial) protein unfolding for barrel passage (17, 47–49). A short amino-terminal motif—referred to as the TonB box—that is part of an unstructured amino-terminal part, is a key segment initiating this transport (23, 50) (Fig. S2) and can be readily retrieved in virtually all ColM-type bacteriocins. Despite sharing only low homologies in their respective RBDs, some other ColM-type pseudocins from the ColMα clade equally take advantage of the ferrichrome transporter, in *Pseudomonas* called FiuA (37). Evolutionary sequence diversification and presumable target sharing by colicin M and (some) ColM-type pseudocins were previously postulated based on prominent structural similarities between the respective bacteriocins (23). Based on high sequence similarities between amino-terminal domains, it is also expected that a certain number of ColMα-type bacteriocins in other enterobacterial genera equally parasitize the ferrichrome receptor. An interesting observation in this context is that homologous ColM pyocins, despite sharing high sequence similarity (90% amino acid identity), do not target the same subset of bacteria (25). Such an altered killing spectrum is likely due to allelic variation in FiuA (51).

In *Pectobacterium* and related genera (*Brenneria* and *Dickeya*), the RBD has been replaced by a ferredoxin module (Pfam PF00111) (21), and for pectocin M1 in particular, piracy of the bacterial plant ferredoxin receptor FusA has been demonstrated (52). As opposed to other ColM bacteriocins, pectocins lack a TonB box for subsequent import, and they possibly depend on FusB (a TonB homologue) for transfer to the periplasm (32, 52). The observation that the integration of a ferredoxin module is detected in only a limited set of taxonomically related organisms suggests that this module was acquired more recently, possibly as a form of niche adaptation. For a distinct ColM-type pyocin retrieved exclusively in *P. aeruginosa* (pyocin M4 [PaeM4], present in strain BL03 and many other isolates), a different receptor is expected since its RBD is significantly longer and lacks sequence similarity with respective domains from FiuA-binding ColM-type pseudocins (Table 1) (25). The same is true for a second cluster of ColMα pseudocins (*Pseudomonas fluorescens* HK44 as a representative [Fig. 1]) and distinct enterobacterial ColM-type bacteriocins from *Erwinia* and *Pantoea*. To date, no receptor has been identified for any of the ColMβ bacteriocins, and it remains to be investigated whether ColMβ bacteriocins all target the same type of receptor.

The piracy of at least two different transporters involved in iron uptake underlines the pivotal role of this metal ion in niche colonization by *Enterobacteriaceae* and other proteobacteria, and a similar strategy is exploited by numerous bacteriocins and phages. At this point, a straightforward prediction of the receptors likely targeted by other ColM-type bacteriocins is complicated by the general lack of known domains (except for the above-mentioned ferredoxin-containing pectocins). Diversifying recombination events have led to a tremendous heterogeneity among nuclease bacteriocins (5, 6, 8, 9, 38); for example, pyocins S1 and S6 share identical amino-terminal regions but are equipped with an HNH-DNase and an rRNase domain, respectively (53). Colicins B and D share a nearly identical amino-terminal region but are equipped with a pore-forming and a tRNase domain, respectively, indicating that recombination events between bacteriocins acting at different cellular levels (cell envelope and cytoplasm) may occur equally (54). Also, in other polymorphic toxin systems, such as contact-dependent growth inhibition (CDI)/antagonism cassettes, glycine zippers, MafB proteins, Rhs effectors, and SitA lipoprotein toxins, recruitment of highly diverse toxin-immunity sets has been noted (18, 55–60). The extent to which such domain shuffling has occurred equally for ColM-type bacteriocins deserves further scrutiny, but the observations that (i) the ferredoxin module has also been recruited to a pesticin domain (32) and (ii) the ColM module has been integrated in at least two hybrid bacteriocin organizations validate ColM as a genuine building block in polymorphic toxins.

### Toxin activation by permissive function.

Following passage through the outer membrane, full-length colicin M requires toxin refolding and activation by the peptidyl-prolyl-*cis*-*trans*-isomerase FkpA (14, 61, 62). This modulating process requires FkpA’s enzymatic activity, although the proline(s) affected has not been identified unambiguously (Fig. S2A) (63). FkpA chaperone processing is probably not a universal characteristic among imported ColM-type bacteriocins, since there are no conserved proline residues present in ColM domains. The residue likely isomerized by FkpA is present only in a number of species taxonomically related to *E. coli* (Fig. S1). In support of that, it was previously suggested that lytic activity of pectocin M1, when expressed in *E. coli* and secreted to the periplasm, does not require FkpA for killing (29). It cannot be excluded, however, that in some species another chaperone(s) provides assistance in activity-generating folding.

Although the actual FkpA contribution in bacteriocin refolding remains partly unsolved, such toxin activation by a permissive factor is not a unique observation. LepB peptidase assists in FtsH-dependent cleavage of colicin D, resulting in release of the tRNase domain, an essential step required for toxin entry into the cytoplasm (64, 65). An *E. coli* tRNase effector delivered to target cells via CDI requires the cysteine biosynthetic enzyme CysK (*O*-acetylserine sulfhydrylase A) for modulation of growth inhibition (66), and RNase activity is displayed only when the toxin is bound to CysK. This toxin stabilization organizes the active site for substrate recognition (67). For other CDI tRNase toxins, GTP-dependent activation by elongation factor Tu (EF-Tu) was detected (68, 69). From the producer’s perspective, activation necessity may seem an undesirable trait, as toxin resistance may develop. For CDI toxins in particular, it was suggested that such activation may play a role in intercellular communication (55): toxins transferred to immune siblings could form heterotrimeric complexes (EF-Tu, CDI toxin, and immunity partner) that influence gene expression and thereby cell-to-cell signaling between clonally related cells (68, 69). Such a toxin exchange mechanism was proposed to initiate biofilm formation in *Burkholderia thailandensis* (70).

### ColM protection provided by an amalgam of immunity partners.

**FIG 2:**
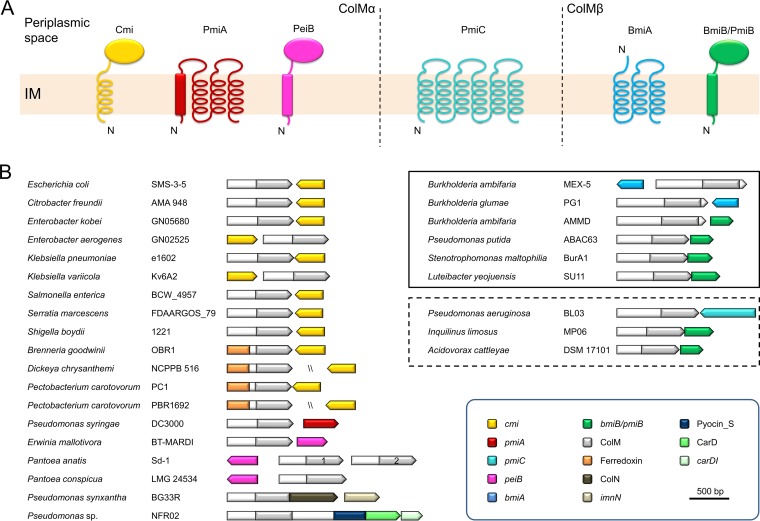
(A) Membrane topology model of characterized and putative ColM-type immunity proteins. Sec/Tat-dependent signal sequences, if predicted in a majority of the cases, are shown as a cylinder instead of a helix. IM, inner membrane; N, amino-terminal end. (B) Schematic gene organization of representative ColM domain-containing bacteriocin and immunity genes (same color as gene products in panel A), including hybrid bacteriocins. The color code specifying different domains and (putative) immunity partners and the scale in base pairs are defined in the key at lower right. In the case of a candidate immunity gene likely presented on a different locus, bacteriocin and immunity genes are separated by two slashes (immunity gene orientation is unknown). ColMβ-type bacteriocins are boxed by a solid line, and ColM-type bacteriocins with unassigned phylogenetic positioning (Fig. 1) are boxed by a dashed line. Imn provides immunity to the ColN pore-forming domain (37), and the carocin D immunity partner (*carDI*) provides immunity to the carocin D (CarD) DNase domain. The ColM-type bacteriocin genes organized in tandem in *Pantoea ananatis* Sd-1 are specified by numerals 1 and 2 (Fig. 1).

The first ColM immunity-providing protein to be identified was Cmi. This 117-aa protein contains a periplasmic YebF-type module and is anchored in the cytoplasmic membrane with an α-helix (Table 1; Fig. 2A) (71–74). The YebF domain is typified by two cysteine residues (75), required for Cmi folding and structure stabilization but not expected to contribute to the immune function *per se*. Based on site-directed mutagenesis of surface-exposed residues, a key role has been proposed for carboxy-terminal residues in Cmi (72). Attempts to copurify and cocrystallize colicin M in complex with Cmi were unsuccessful; therefore, it remains elusive how silencing of the ColM activity domain is actually achieved (71). Candidate *cmi* orthologues with a YebF signature can be readily retrieved in the vast majority of the enterobacterial isolates hosting ColMα-type bacteriocin genes (Fig. 1). As for *E. coli*, most *cmi*-like immunity genes are located downstream of the bacteriocin gene, in the opposite direction (Fig. 2B). Occasionally, *cmi* may also be located upstream, as, e.g., in *Klebsiella variicola* Kv6A2 and *Enterobacter aerogenes* GN02525, which is rather atypical for polymorphic toxin organizations in general and likely results from recombination events. In most pectobacteria, a *cmi* homologue is genomically separated from the pectocin M gene (21), presumably resulting from genome rearrangements. However, the potential role of the encoded protein in providing pectocin immunity remains to be explored.

Interestingly, pseudomonads do not carry *cmi*-like genes in their genomes (22). Instead, immunity to bacteriocins of the ColMα clade is provided by a different type of protein, PmiA, adopting a four-transmembrane-helix (TMH) topology (Fig. 2A) (24), an architecture that one would associate rather with immunity proteins for pore-forming bacteriocins (6, 8, 76). PmiAs are encoded downstream of the ColM-type pseudocin genes and essentially do not show sequence conservation, except for a short semiconserved motif that is predicted to be exposed to the periplasm. When mutated within this motif, PmiA was found to still exert its function (24). Taking into account that Cmi remains active in the periplasm when deprived of its membrane anchor (77), PmiA likely shows immunity at a different cellular location, i.e., in the cytoplasmic membrane. Yet another candidate immunity partner is expected for ColMαs from *Pantoea* and *Erwinia* (PeiB, *Pantoea-Erwinia* ColM-type immunity type B). These bacteriocin genes are associated (upstream and downstream, respectively) with a gene encoding an immunity protein with a similar topology as Cmi but lacking the YebF signature domain.

The majority of the ColMβ-type bacteriocin genes are associated with an immunity partner adopting a similar topology as Cmi and PeiB, termed BmiB and PmiB in *Burkholderia* and *Pseudomonas*, respectively (20, 24). Their periplasmic module again lacks the YebF signature domain, cannot be meaningfully aligned with Cmi or PeiB, and therefore possibly represents a third type of periplasmic ColM immunity domain. The presence of two strictly conserved cysteines may point to disulfide bond formation, as observed for Cmi (71, 72). Other ColMβ bacteriocins from *Stenotrophomonas* and *Luteibacter* are equally associated with a putative *bmiB*/*pmiB*-type immunity gene, residing downstream on the same strand (Fig. 2A and B). The encoded proteins are equipped with two cysteines as well, although obvious sequence similarity with BmiBs and PmiBs is present only in the carboxy-terminal part of these candidate immunity proteins. A small set of ColMβ-type burkhocins are associated (upstream or downstream) with a three-TMH immunity partner, BmiA (20), reminiscent of the PmiA organization for ColMα bacteriocins in pseudomonads. Given that ColMβ-BmiA organizations are rare and display close clustering (Fig. 1), one may speculate that this immunity gene was acquired more recently during evolution. The candidate immunity partner of the distinct ColM-type pyocin from *P. aeruginosa* BL03 (PmiC), encoded downstream in the opposite direction, adopts yet another organization with six TMHs (Fig. 2).

### The ColM family: fitting in the toxin-immunity coevolution model?

The observation that the ColM domain has recruited different unrelated immunity genes is surprising but does not necessarily argue against the toxin-immunity coevolution model of diversifying selection. In this model, a first mutation in an immunity gene results in a broadened immunity function. Bacteriocin producers are now immune to the ancestral bacteriocin but also have gained immunity to other similar toxin domains, which is interesting from the producer’s point of view. A second mutation in the toxin module may follow and does not necessarily have a negative impact on the diverged immunity gene (78–80). Cells that do not host the new immunity gene are rapidly outcompeted in this scenario, a process that keeps on repeating itself and eventually results in diverged toxin families protected by their own cognate immunity gene. At this point, there is no clear explanation why a multitude of immunity proteins have been recruited by ColM-type bacteriocins, and such a phenomenon has not been noticed for other polymorphic toxins so far. In Gram-positive bacteria, a similar observation has been made for the peptidoglycan-degrading bacteriocin 41 from *Enterococcus faecalis*: two distinct immunity proteins have been described for this peptidoglycan endopeptidase, equipped with two or four TMHs (81).

A number of options can be considered. (i) Part of the answer will likely be provided by the interaction between the ColM domain and its chaperone(s) and the structural consequences thereof. At this point, it cannot be excluded that ColMs (in different genera/clusters) require different permissive factors. This is a reasonable assumption considering the absence of a universally conserved proline in ColM domains (see above). The interacting chaperone(s) may ultimately define which type of immunity partner is required. (ii) Different immunity protein topologies may point to immunity at different cellular locations, i.e., fundamentally different mechanisms would exist to impede lipid II-degrading activity, targeting different patches of the ColM domain for interaction, directly or indirectly impeding the enzymatic function. Considering that integral membrane proteins in the ColMα group arise only in pseudomonads and a small subset of *Burkholderia* strains (ColMβ), this may suggest that immunity by this type of protein was obtained later in evolution. (iii) The observations that the key residues for Cmi immunity are located at the carboxy terminus of the protein and that PmiAs sharing very poor sequence similarity with the cognate immunity protein may provide noncognate immunity (24, 72) could result in a projective interpretation of the relevance of immunity protein topology. Possibly, only a small patch is critical for immunity. (iv) From an evolutionary point of view, the pressure to cotranscribe an immunity gene together with a ColM-type bacteriocin is generally high and required *per se*. Given that the genomic positioning of ColM bacteriocins is very diverse, even within a certain genus, genetic coupling with a novel immunity partner may be the fortuitous consequence of a seemingly unsuccessful recombination event. In this scenario, ColM immunity was temporarily provided at a different locus, and in a later phase replaced by a neofunctionalized bacteriocin-adjacent gene. This hypothesis may explain why hybrid ColM-type bacteriocins exist and does not necessarily argue against the classical interpretation of the toxin-immunity coevolution model, since diversifying selection may still occur. (v) As lipid II tends to appear in regions of increased fluidity (RIFs), hereby forming “atoll”-like regions in the inner membrane, different immunity protein topologies may be the result of preferential (genus-dependent) association or integration in these areas to protect lipid II from enzymatic digestion (16).

It will also be of future interest to explore whether kin selection is involved in ColM-immunity divergence. This model states that genealogical relatives have more alleles in common than nonrelated strains; therefore, the probability of maintaining a gene in subsequent generations will increase if it is associated with an enhanced fitness for the genealogical relatives (55). Toxin-immunity modules, especially those for which immunity is highly selective, as present in (certain) colicins and CDI cassettes, have been previously studied in this context (55, 82–84).

### Concluding remarks.

Continued scrutiny of the *E. coli* ColM system along with exploration of related systems from other proteobacterial genera has revealed ColM’s catalytic site and noncatalytic activity, allowed delineation of structural domains that may be the target of diversifying recombination and selection events, dissected the contribution of a first permissive factor, and revealed a whole array of topologically distinct membrane-associated immunity proteins. The features emerging from this broadened view are indicative of a genuine polymorphic toxin family and a patchwork of immunity partners, giving rise to several new scientific questions and hypotheses. The essentiality of lipid II for cell viability makes ColM bacteriocins an attractive candidate drug worth exploring further.
