# Supplementary figures and images for: The ColM Family, Polymorphic Toxins Breaching the Bacterial Cell Wall
Source: mBio. 2018 Feb 13;9(1):e02267-17. doi: 10.1128/mBio.02267-17 (PMC5821083; doi:10.1128/mBio.02267-17)

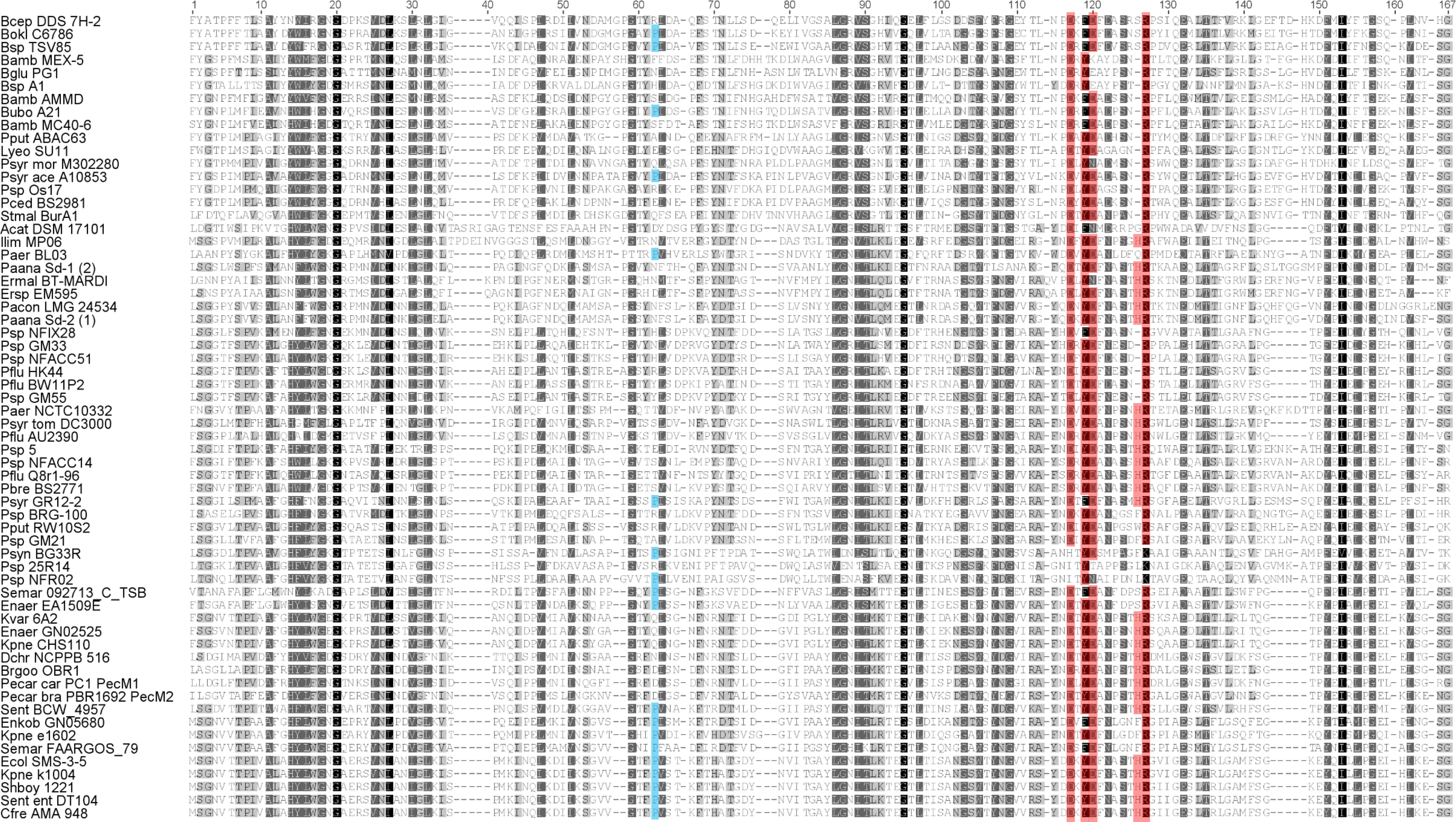

Supplement: FIG S1 [file mbo001183723sf1.tif]

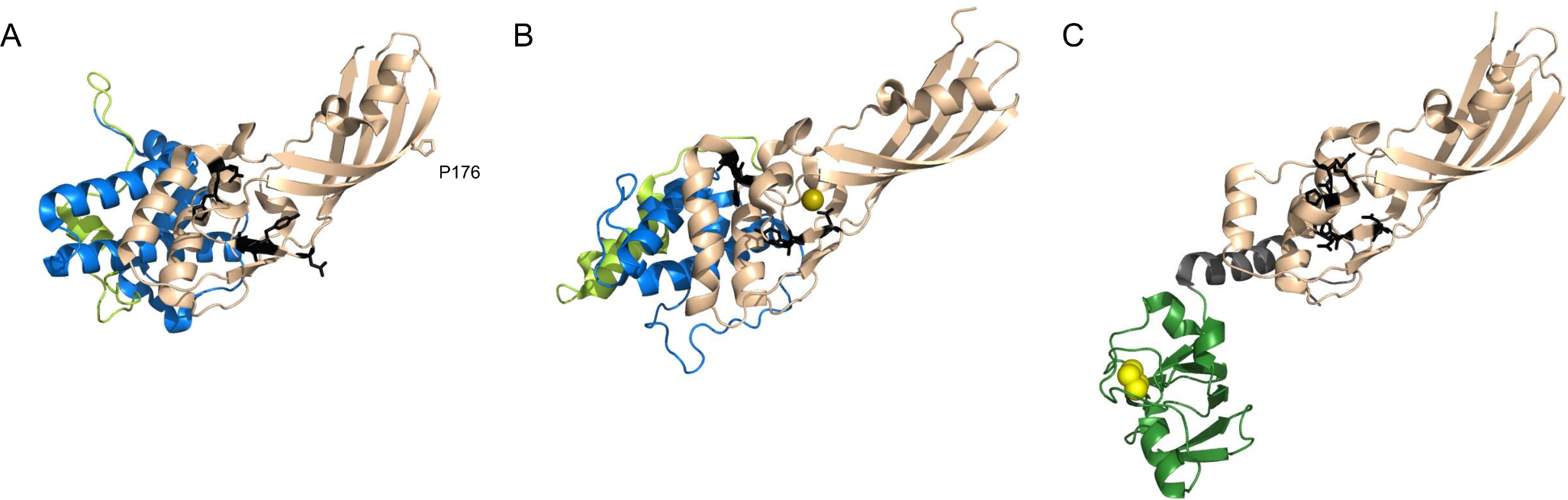

Supplement: FIG S2 [file mbo001183723sf2.tif]
